# Hospital Smoke-Free Policy: Compliance, Enforcement, and Practices. A Staff Survey in Two Large Public Hospitals in Australia

**DOI:** 10.3390/ijerph14111358

**Published:** 2017-11-08

**Authors:** Sam McCrabb, Amanda L. Baker, John Attia, Zsolt J. Balogh, Natalie Lott, Kerrin Palazzi, Justine Naylor, Ian A. Harris, Christopher M. Doran, Johnson George, Luke Wolfenden, Eliza Skelton, Billie Bonevski

**Affiliations:** 1 School of Medicine and Public Health, Faculty of Health and Medicine, University of Newcastle, Callaghan, New South Wales 2308, Australia; Amanda.Baker@newcastle.edu.au (A.L.B.); John.Attia@newcastle.edu.au (J.A.); Zsolt.Balogh@hnehealth.nsw.gov.au (Z.J.B.); Luke.Wolfenden@hnehealth.nsw.gov.au (L.W.); Eliza.Skelton@newcastle.edu.au (E.S.); Billie.Bonevski@newcastle.edu.au (B.B.); 2 Hunter Medical Research Institute, University of Newcastle, New Lambton, New South Wales 2305, Australia; Kerrin.Palazzi@hmri.org.au; 3 Department of General Medicine, John Hunter Hospital, New Lambton Heights, New South Wales 2305, Australia; 4 Department of Traumatology, John Hunter Hospital, New Lambton Heights, New South Wales 2305, Australia; Natalie.Lott@hnehealth.nsw.gov.au; 5 Whitlam Orthopaedic Research Centre, Ingham Institute for Applied Medical Research, Liverpool Hospital, Liverpool, New South Wales 2170, Australia; Justine.Naylor@sswahs.nsw.gov.au (J.N.); iaharris1@gmail.com (I.A.H.); 6 South Western Sydney Clinical School, Faculty of Medicine, University of New South Wales, Liverpool, New South Wales 2170, Australia; 7 School of Human, Health and Social Sciences, Central Queensland University, Brisbane, Queensland 4000, Australia; C.Doran@cqu.edu.au; 8 Centre for Medicine Use and Safety, Monash University, Melbourne, Victoria 3052, Australia; Johnson.George@monash.edu; 9 Hunter New England Population Health, Wallsend, New South Wales 2287, Australia

**Keywords:** smoking cessation care, tobacco control, smoke-free policy

## Abstract

**Background:** Smoke-free hospital policies are becoming increasingly common to promote good health and quit attempts among patients who smoke. This study aims to assess: staff perceived enforcement and compliance with smoke-free policy; the current provision of smoking cessation care; and the characteristics of staff most likely to report provision of care to patients. **Methods:** An online cross-sectional survey of medical, nursing, and allied staff from two Australian public hospitals was conducted. Staff report of: patient and staff compliance with smoke-free policy; perceived policy enforcement; the provision of the 5As for smoking cessation (Ask, Assess, Advise, Assist, and Arrange follow-up); and the provision of stop-smoking medication are described. Logistic regressions were used to determine respondent characteristics related to the provision of the 5As and stop-smoking medication use during hospital admission. **Results:** A total of 805 respondents participated. Self-reported enforcement of smoke-free policy was low (60.9%), together with compliance for both patients (12.9%) and staff (23.6%). The provision of smoking cessation care was variable, with the delivery of the 5As ranging from 74.7% (ask) to 18.1% (arrange follow-up). Medical staff (odds ratio (OR) = 2.09, CI = 1.13, 3.85, *p* = 0.018) and full time employees (OR = 2.03, CI = 1.06, 3.89, *p* = 0.033) were more likely to provide smoking cessation care always/most of the time. Stop-smoking medication provision decreased with increasing age of staff (OR = 0.98, CI = 0.96, 0.99, *p* = 0.008). **Conclusions:** Smoke-free policy enforcement and compliance and the provision of smoking cessation care remains low in hospitals. Efforts to improve smoking cessation delivery by clinical staff are warranted.

## 1. Introduction

Tobacco smoking is one of the leading preventable causes of morbidity and mortality, leading to the death of approximately 6 million people each year [1]. Acknowledging the key role they may play in promoting smoking cessation, hospitals worldwide are becoming increasingly smoke-free [2]. International organisations exist to support the movement towards whole of hospital smoking bans (e.g., the International Network of Health Promoting Hospitals and Health Services [2] and the ENSH-Global Network for Tobacco Free Healthcare Services) [3]. This is important, as hospital admission has often been cited as being a time and place when individuals are open to health advice, such as advice on smoking cessation [4].

Understanding levels of compliance (i.e., patients and staff abiding by the policy) and enforcement (i.e., staff ensuring the policy directives are followed by patients and other staff) are measures often used to determine policy implementation [5]. Determining which factors are associated with the current provision of care, and staff differences in the provision of care, is essential to better target interventions to increase the effectiveness of hospital smoke-free policy [6]. Lavis et al. [7] highlight five key questions which can assist in identifying where interventions for implementing policy change are needed: (1) What is lacking? (2) To whom is it lacking? (3) Who needs to drive change? (4) How should change occur (what barriers are there)? and (5) With what effect has change been successfully implemented? In relation to hospital smoking cessation policy, this indicates that there is a need to understand what current practice is and by whom it is being provided in order to highlight where interventions need to be targeted and who should deliver them. After determining these factors, the implementation of new interventions can then be planned based on the identified barriers and facilitators to current practice.

In order to support smoking cessation attempts, legislation for the progression toward smoke-free hospitals in New South Wales (NSW), Australia, has been in effect for many years (1999). The initial implementation of whole smoke-free campuses had been planned to take effect in September 2002; however, it was not until 2015 that the *NSW Health Smoke-free Health Care Policy 2015* was instigated, designating all hospital buildings, vehicles, and grounds as completely smoke-free locations, and requiring people to leave the hospital grounds to smoke [8]. This policy recommends the provision of smoking cessation care using the 5As approach [9]: Ask patients about their tobacco smoking; Assess their levels of use; Advise the individuals that they should stop smoking; Assist them to make or plan a quit attempt; and Arrange follow-up with hospital staff or another stop smoking service/individual. The provision of Nicotine Replacement Therapy (NRT) during admission that continues for at least three days post-discharge, along with a referral to the state telephone Quitline, are also recommended.

Despite the implementation of smoke-free legislation, the provision of smoking cessation care remains low. In 2008, a meta-analysis of patient reported receipt of care found that only 60% of individuals report having their smoking status assessed, with 42% being advised or counselled to quit, 14% provided with NRT, and 12% receiving a referral or follow-up [10]. Freund et al. also found that staff-reported provision of care, although slightly higher, was still suboptimal [10]. More recently, a 2015 review of NSW public hospitals’ patient files reported significant gains in the provision of smoking cessation care over a four-year period (2005–2009). Nevertheless, only just over half of patients received smoking cessation care (advice to quit, an offer of NRT, or an offer for a Quitline referral) [11]. Over 10 years have passed since the initial implementation of the hospital smoke-free policy, and it is important to measure progress towards policy adoption and the current practices of medical staff in order to gauge implementation success.

This study aims to assess the compliance with and enforcement of hospital smoke-free policy, and provision of current hospital smoking cessation care. It also aims to examine if the provision of smoking cessation care differs based on staff characteristics.

Specifically, this study aims to:

(1) assess staff perceived compliance with and enforcement of hospital smoke-free policies;
(2) assess staff practices regarding the provision of smoking cessation care; and
(3) examine the characteristics of staff associated with the provision of smoking cessation care to hospitalised patients.

## 2. Materials and Methods

### 2.1. Design and Setting

The study consisted of an online cross-sectional survey of employees at two public hospitals in NSW, Australia. Surveys were conducted over a three-month period: March–September 2016. Both hospitals are Level 1 trauma facilities, with over 500 beds and an emergency department. Neither of these hospitals were members of a global network for tobacco control, but they were covered by state hospital policy and current clinical recommendations.

### 2.2. Participants

Current employees of the hospitals with patient contact for therapeutic reasons were eligible to participate in the survey. Administration and ancillary staff were excluded as it was not in their role to provide medical support to patients.

Study contacts at both hospitals were asked to send invitation emails (containing a participant information statement and a link to the survey) to employees using Human Resource databases. Two reminder emails were sent during the data collection phase, the first at one month and the second at two months following the initial invitation email. A screening question was included at the start of the survey asking *‘In your role at the hospital, do you have patient contact for therapeutic reasons?’* (Yes; No). Those individuals who answered ‘No’ were excluded from survey participation. All surveys were completed anonymously, with commencement regarded as consent. At Hospital 1, all hospital staff received the invitation email, and in Hospital 2, only medical and nursing staff received the invitation email; this was at the discretion of the hospital’s administrators.

### 2.3. Measures

Where possible, existing measures were used, adapted, or generated from the literature [9,11,12,13,14]. The items reported in this study were part of a larger survey which took participants an average of 7.7 min to complete. Supplementary Table S1 lists the survey items.

*Staff characteristics.* Age, gender, role at hospital, duration of hospital employment, current smoking status [12], and intentions to quit [13] were measured.

*Enforcement.* To determine perceived smoke-free policy enforcement, respondents were asked to indicate how well hospital policy was enforced on a six-point Likert-scale (Always; Often; Sometimes; Rarely; Never; Unsure). Using a seven-point Likert-scale (Always; Often; Sometimes; Rarely; Never; Unsure; Not applicable), staff were asked to determine how often they asked other staff/patients to stop smoking or requested other staff/patients to leave the hospital premises for smoking. Respondents were also asked to indicate if they offered NRT to a patient when they saw them smoking on campus.

*Compliance.* Staff were asked to answer the questions *‘How often do patients from your ward who smoke adhere to the smoking restrictions at the hospital?’* and *‘How well are the hospital smoking restrictions adhered to by staff? That is, for staff members who smoke, how well do they adhere to the hospital no smoking policy?’* using a six-point Likert-type scale (Always; Often; Sometimes; Rarely; Never; Unsure).

*Provision of smoking cessation care.* Using a four-point Likert-type scale (Always; Most of the time; Rarely; Never), respondents were asked to indicate how often they provided the 5As of smoking cessation (asked individuals if they smoked, advised individuals to quit, assessed current smoking status, assisted in making a quit plan, and arranged a follow-up) as well as provide a referral to Quitline or other specialist stop-smoking services. Respondents were also asked to indicate *‘How is the decision concerning whether or not to provide hospitalised patients who smoke with assistance to quit made?’* (Assistance is offered to every patient who smokes; Assistance is offered on a patient-by-patient basis; Assistance is offered only if the patient requests it; Unsure) to determine if there was selectiveness in how care was provided.

### 2.4. Statistical Analysis

All data was stored on secure servers at the University of Newcastle and was exported into STATA v13 (Statacorp LP., College Station, TX, USA) for analysis. Descriptive statistics are presented as numbers and percentages for categorical variables, and means (standard deviation; SD) or median (quartile 1, quartile 3) for continuous variables, depending on the distribution of the data.

Binary logistic regression models were used to examine respondent characteristics (age, gender, role at hospital, employment, and smoking status) related to the provision of care and the offer of stop-smoking medication during admission. Individuals who indicated either ‘always’ or ‘often’ to the provision of all of the 5As questions (Ask them about their current level of use; Advise them to quit; Assess their willingness to quit; Assist them with a quit plan; Arrange follow-up with them either yourself or another service) were categorised as having provided the 5As ‘yes’; otherwise, they were categorised as ‘no’. The provision of stop-smoking medication was determined by combining the measures ‘offer Nicotine Replacement Therapy’ and ‘offer them other pharmacotherapies to help them quit’, with individuals who indicated either ‘always’ or ‘often’ categorised as ‘yes’. Variables entered in the regression were selected a priori based on literature: age; gender; role at hospital; position; and smoking status [15,16,17,18,19]. Collinearity of variables related to smoking cessation care was checked using variance inflation factors (VIFs). No variables were found to be collinear, with all VIFs less than two. Crude and adjusted odds ratios (OR) with 95% confidence intervals (CI) and *p*-values were calculated for variables in the models. Significance was determined at *p* < 0.05.

## 3. Results

### Participants

A total of 7023 individuals (6266 from Hospital 1 and 757 from Hospital 2) were sent an email to participate in the online survey. Of the 1261 individuals who commenced the survey (1158 from Hospital 1 and 103 from Hospital 2)—providing a combined response rate of 18%—456 were ineligible due to a lack of patient contact. A total of 805 eligible participants took part (712 from Hospital 1 and 93 from Hospital 2). Individuals were able to withdraw from participation at any time point. For this reason, the total number of respondents for each survey item may differ. A total of 647 individuals completed the survey, providing a completion rate of 80%.

*Participant characteristics.* Participant demographics can be found in Supplementary Table S2. The majority of respondents were female (79.9%), with a mean age of 42.5 (SD = 12.3). More than half identified as registered or enrolled nurses (53.7%), 21.7% identified as medical doctors, and 24.7% identified as other allied health personnel. The majority of respondents were employed full-time (61.7%) and had worked at the hospital for 10 or more years (50.9%). Only 5.6% of participants identified as current smokers.

*Enforcement.* The majority of respondents indicated that the total smoking ban was enforced rarely/never (60.9%). Few respondents indicated that they would always/often ask another staff member to stop smoking if seen smoking (5.6%) or to leave the hospital premises to smoke (7.1%). Only 9.8% of staff indicated that they would always/often ask patients to stop smoking or to leave the hospital premises (11.7%). Twenty seven percent of respondents indicated they would offer NRT to a patient found to be smoking on campus (Table 1).

*Compliance.* Respondents indicated that few of their patients always/often adhered to restrictions on their ward (12.9%). However, staff indicated that the rate of staff adherence to smoking policy was higher, with 23.6% of staff who were current smokers adhering to hospital policy always/often (Table 1).

*Provision of smoking cessation.* The provision of smoking cessation is summarised in Table 2. For the 5As (Ask, Advise, Assess, Assist, and Arrange), provision ranged from 74.7% of respondents reporting that they would ask patients about their level of use to 18.1% indicating they would arrange a follow-up (Table 2). This was dichotomised to reflect the groupings used in the logistic regression. Referral to another service for smoking cessation support and the offer of stop-smoking medication was also low.

*Assisting patients to quit.* When asked how the decision concerning whether to provide hospitalised patients help to quit was made, 36.3% were unsure, 32.3% provided assistance to every patient who smoked, 26.1% offered assistance on a patient-by patient basis, and 5.3% indicated they only offered assistance to patients who requested it (Table 3).

*Characteristics of respondents likely to provide care.* A total of 87 individuals stated that they would provide the 5As of smoking cessation always/most of the time. The odds of providing the 5As were 2.09 times higher among medical staff (intern, resident, registrar, or consultants) than nurses (95% CI = 1.13, 3.85, *p* = 0.018). Further, the odds of providing the 5As were 2.03 times higher if the individual was a full-time employee compared to less than full-time employees (part time, casual, other) (95% CI = 1.06, 3.89, *p* = 0.033) (Table 4).

A total of 144 staff members indicated that they would provide smoking cessation medication always/most of the time. When examining respondent characteristics related to the provision of stop-smoking medication (either NRT or other pharmacotherapies) always/most of the time, only staff age was found to be significant, with a 2% reduction in the odds of the provision of stop-smoking medication for each 1 year age increase (OR = 0.98, 95% CI = 0.96, 0.99, *p* = 0.008) (Table 4).

## 4. Discussion

While gains have been made to increase compliance with and the enforcement of smoke-free hospital policies and the provision of smoking cessation care, further improvement is needed. Consistent with previous studies conducted among Australian hospitals [11,20,21], these results indicate that the provision of care is still suboptimal and suggest that little progress has been made in the last decade. Given the negative impact continuing tobacco use can have on recovery [22,23], length of hospital admission, and complications [24], continuing to addressing tobacco smoking in the hospital setting should be a priority.

A possible reason for low compliance with hospital smoke-free policy by patients may be a lack of patient awareness of the policy, which has been previously identified [21], or difficulties with compliance and enforcement, which has also been found previously [5]. One potential solution to a lack of patient awareness may include patient education upon admission, potentially in the form of a short film including information about why smoking is banned; the links between continued tobacco use and delayed wound healing and complications [23,25,26]; increased cost to individuals and the health system [24]; and the public health impact on others [27]. Such education should be accompanied by the provision of NRT and counselling assistance or access to Quitline while in hospital.

Developing an organisational structure which is responsible for the enforcement of hospital policy may provide the systematised nature necessary for taking the final steps towards 100% smoke-free campuses and improving compliance. Certainly, organisational change may help to provide a structure for staff to follow with regards to the provision of smoking cessation care if they feel that they lack the education or training necessary to provide smoking cessation support [28]. Previous research has indicated that organisational structure, and the nature of a ‘chain of command’ (i.e., a top down approach), is helpful in policy implementation as it provides guidance as to who is responsible. However, all levels of an organisation need to be involved in the process of change and policy implementation to ensure staff buy-in [29]. Passive smoke-free policy implementation is not sufficient to ensure successful implementation, with a number of strategies identified as promoting policy sustainability [30]. Engaging staff and promoting the importance of addressing tobacco smoking with their patients and the benefits cessation can have on both patient and service outcomes is essential to increasing the provision of care.

It cannot be overlooked that three quarters of staff indicated that they would rarely or never ask another staff member or patient to stop smoking or leave the hospital’s grounds. This may be out of concern for their own personal safety, an important factor to acknowledge especially given the high rates of staff assault that has been identified [31]. However, it was suggested by a recent review of Australian nurses that the introduction and enforcement of a zero tolerance policy reduces violence and improves safety, further indicating that patient violence was most often the result of long waiting times [32]. Internationally, it has also been found that the implementation of a smoking ban decreases physical violence [33]. While these are both measures of inpatient smoking, there is little evidence regarding levels of violence on the campus (that is, outside of hospital buildings), an area which may be regarded as separate to the hospital admission area, beyond the location of where therapeutic relationships exist between medical professionals and patients/visitors. It may, therefore, be the role of security guards to provide smoke-free policy enforcement outside of hospital buildings. However, issues such as security guards themselves often being seen smoking on hospital grounds, inconsistency among security guards regarding ignoring and enforcing the policy [5], and that allowing patients to smoke has been used as a bargaining chip to get patients to behave as desired [34] have been identified. Education and training may be one way to address this and increase policy enforcement on campus. Additionally, given that a low percentage (27.3%) of staff indicated that they would provide patients who smoke with NRT, there is potential to use this approach to provide smoking cessation care to individuals in a non-confrontational manner. Further staff training would be needed to educate staff about the types of NRT, their uses, and ways to have conversations with patients when offering the medication.

Applying the questions proposed by Lavis et al. [7], and looking specifically at what care is being provided and by whom, it was found that medical staff were more likely to provide smoking cessation care than nursing staff. Similar results have found that nursing staff are often unlikely to provide smoking cessation support [35,36]. That nursing staff report providing care less than other medical staff may reflect different barriers felt by nursing staff, such as that it is not their role [36]; a lack of confidence, inadequate training, or a lack of time and resources [19,35,36,37]. Addressing these barriers and how they affect nursing staff may assist in the development of any future intervention to increase the provision of care by this group.

Considering that patient contact with nursing staff is high [38], improving nurse-led smoking cessation care may be one way to increase the provision of smoking cessation care received by patients. Various studies have examined this, with education found to be effective at increasing nurse-led intervention [39,40]. Further education in medical and nursing university courses may be needed to emphasise the importance of smoking cessation care, together with the implementation of a refresher training system to promote the provision of smoking cessation care. This may be one of the ways to drive change addressing Lavis et al.’s [7] third question (Who needs to drive change?), with universities identified as one of the messengers for knowledge transfer. While this may offer a potential solution to the problem of low provision of care, there is acknowledgement that this may present feasibility issues regarding costs involved with organising further education. Alternatively, integrating smoking cessation care into the responsibilities of an already existing team or organisation, for example the hospital’s drug and alcohol team, may be one way to overcome provision of care limitations. Similar success utilising this method has been found in the drug and addiction treatment field [41,42]; however, in the hospital setting, this may be hampered by barriers to care, such as a ‘lack of time’ [19,35,36,37] or referral issues.

Equipping staff with the tools to reduce the demands on their time may present another possible solution to addressing care provision. The presence of a smoking cessation counsellor or referral to drug and alcohol clinics in the hospital may help, although this presents feasibility issues due to funding. Alternatively, technology-based interventions may represent the future of health care, with online smoking cessation programs found to increase quit attempts [43,44,45]. However, there may be difficulties regarding the development of such an intervention due to the diverse needs of different patient population groups, with further investigation needed.

## 5. Limitations

The study’s main limitation is the low response rate. The response rates to surveys of health care providers are notoriously low [46,47,48], and ours is typical, especially of organisation-wide surveys where it is hard to target eligible individuals. Further, data on non-respondents was unable to be collected, and therefore this data may underrepresent some respondent groups. Additionally, it may be that the staff who responded to the survey are likely to be more interested in the topic of smoking, hence biasing the sample. Despite this, a strength of the study is the broad range of hospital-based professions captured from two of the state’s largest hospitals. Further, this study takes a unique approach in determining the success of hospital smoke-free policy implementation from staff member’s perspectives and highlights where intervention to increase the provision of care needs to be focused.

## 6. Conclusions

Despite difficulties associated with smoke-free hospital policy implementation, the provision of smoking cessation care and the enforcement of and compliance with these policies remains low. It is integral that interventions to increase the provision of care and the enforcement of hospital policy are found. Increasing the training received by staff, changing the culture around who is responsible for providing care, or developing an intervention which bypasses some of the staff barriers to providing care may offer a solution to this issue. Nurse-specific training, increasing staff collaboration with the hospital’s drug and alcohol or security teams, the inclusion of an onward smoking cessation counsellor, or the development of an online intervention may be potential techniques which can be utilised to increase the provision of care. Further research looking at staff barriers and potential factors which may facilitate smoke-free policy implementation needs to be conducted in order to guide policy implementation, ensure staff buy-in, and highlight who should drive change.

## Figures and Tables

**Table 1:** Enforcement of hospital smoke-free policy and perceived compliance with hospital smoke-free policy.

	Total (*n* = 777) *n* (%)				
	Always/Often	Sometimes	Rarely/Never	Unsure	Not Applicable
How often is the total smoking ban enforced at your hospital?	122 (15.7%)	135 (17.4%)	473 (60.9%)	47 (6.1%)	-
How often do patients from your ward who smoke adhere to the smoking restrictions at the hospital?	100 (12.9%)	172 (22.2%)	429 (55.4%)	74 (9.6%)	-
How well are the hospital smoking restrictions adhered to by staff? That is, for staff members who smoke, how well do they adhere to the hospital no smoking policy?	183 (23.6%)	203 (26.3%)	298 (38.5%)	88 (11.4%)	-
If you see a staff member smoking on campus, how often do you:	**Total (*n* = 770)** ***n* (%)**				
Ask them to stop smoking?	43 (5.6%)	72 (9.4%)	572 (74.3%)	4 (0.5%)	79 (10.3%)
Ask them to go outside the hospital premises for smoking?	55 (7.1%)	63 (8.2%)	556 (72.2%)	5 (0.7%)	91 (11.8%)
If you see a patient smoking on campus, how often do you:	**Total (*n* = 768)** ***n* (%)**				
Ask them to stop smoking?	75 (9.8%)	92 (12.0%)	589 (76.7%)	1 (0.1%)	11 (1.4%)
Ask them to go outside the hospital premises for smoking?	90 (11.7%)	80 (10.4%)	571 (74.3%)	2 (0.3%)	25 (3.3%)
Offer them Nicotine Replacement Therapy instead of smoking?	210 (27.3%)	126 (16.4%)	376 (48.6%)	4 (0.5%)	52 (6.8%)

**Table 2:** Provision of smoking cessation care.

When Talking to a Patient Who Had Identified as a Current Smoker, How Often Do You:	Total (*n* = 707)	
	Rarely/Never *n* (%)	Always/Most of the Time *n* (%)
Ask them about their current level of use	179 (25.3%)	528 (74.7%)
Advise them to quit	203 (28.7%)	504 (71.3%)
Assess their willingness to quit	250 (35.4%)	456 (64.6%)
Assist them with a quit plan	436 (61.8%)	269 (38.2%)
Refer them to specialist stop-smoking services or counsellor	549 (77.9%)	156 (22.1%)
Refer them to telephone Quitline	511 (72.5%)	194 (27.5%)
Offer Nicotine Replacement Therapy	281 (39.8%)	426 (60.3%)
Offer them other pharmacotherapies to help them quit	560 (79.4%)	145 (20.6%)
Provide post-discharge help to quit	574 (81.3%)	132 (18.7%)
Arrange follow-up with them either by yourself or another service	578 (81.9%)	128 (18.1%)

**Table 3:** How the decision to provide smoking cessation care is made.

In Your Hospital, How Is the Decision Concerning Whether or Not to Provide Hospitalised Patients Who Smoke with Assistance to Quit Made?	Total (*n* = 697) *n* (%)
Assistance is offered to every patient who smokes	225 (32.3%)
Assistance is offered on a patient-by-patient basis	182 (26.1%)
Assistance is offered only if the patient requests it	37 (5.3%)
Unsure	253 (36.3%)

**Table 4:** Logistic regression of variables related to the provision of 5As often or always, and offer of stop-smoking medication.

**The Provision of 5As Often or Always ^**	**Yes** ***n* (%)**	**Crude**		Adjusted *	
		**Odds Ratio (95%)**	***p*-Value**	**Odds Ratio (95%)**	***p*-Value**
Age		1.00 (0.98, 1.02)	0.840	1.00 (0.98, 1.02)	0.946
Gender			0.240		0.859
Male	20 (15.4%)	Ref.		Ref.	
Female	60 (11.6%)	0.72 (0.42, 1.25)		0.94 (0.49, 1.82)	
Role at hospital			0.001		0.018
Nurses	39 (10.1%)	Ref.		Ref.	
Medical staff	33 (21.6%)	2.44 (1.47, 4.05)		2.09 (1.13, 3.85)	
Employment			0.066		0.033
Other	25 (9.4%)	Ref.		Ref.	
Full time	62 (14.1%)	1.59 (0.97, 2.59)		2.03 (1.06, 3.89)	
**Offer of Stop-Smoking Medication during Admission ^#^**	**Yes** ***n* (%)**	**Crude**		Adjusted *	
		**Odds Ratio (95%)**	***p*-Value**	**Odds Ratio (95%)**	***p*-Value**
Age		0.98 (0.97, 1.00)	0.032	0.98 (0.96, 0.99)	0.008
Gender			0.783		0.233
Male	27 (20.8%)	Ref.		Ref.	
Female	102 (19.7%)	0.94 (0.58, 1.51)		0.71 (0.40, 1.25)	
Role at hospital			0.959		0.071
Nurses	86 (22.3%)	Ref.		Ref.	
Medical staff	34 (22.1%)	0.99 (0.63, 1.55)		0.60 (0.35, 1.04)	
Employment			0.116		0.121
Other	46 (17.3%)	Ref.		Ref.	
Full time	98 (22.2%)	1.37 (0.93, 2.02)		1.47 (0.90, 2.38)	

* Adjusted for age, gender, role at hospital, employment, and smoking status; ^ The variables ‘Ask them about their current level of use’; ‘Advise them to quit’; ‘Assess their willingness to quit’; ‘Assist them with a quit plan’; and ‘Arrange follow-up with them either yourself or another service’ were combined to form this variable; ^#^ The variables ‘offer Nicotine Replacement Therapy’ and ‘offer them other pharmacotherapies to help them quit’ were combined to form this variable.

## Supplementary Materials

The following are available online at www.mdpi.com/1660-4601/14/11/1358/s1, Supplementary Table S1: Survey items. Supplementary, Table S2: Sociodemographic characteristics of sample. Supplementary data file: csv file of participant responses.

## References

[1] World Health Organization Tobacco. http://www.who.int/mediacentre/factsheets/fs339/en/.

[2] Pelikan J.M., Krajic K., Dietscher C. (2001). The health promoting hospital (HPH): Concept and development. Patient Educ. Couns..

[3] ENSH Global Network for Tobacco Free Health Care Services. http://www.ensh.org/.

[4] Dohnke B., Ziemann C., Will K.E., Weiss-Gerlach E., Spies C.D. (2012). Do hospital treatments represent a ‘teachable moment’ for quitting smoking? A study from a stage-theoretical perspective. Psychol. Health.

[5] Schultz A.S., Finegan B., Nykiforuk C.I., Kvern M.A. (2011). A qualitative investigation of smoke-free policies on hospital property. Can. Med. Assoc. J..

[6] Bloor R., Meeson L., Crome I. (2006). The effects of a non-smoking policy on nursing staff smoking behaviour and attitudes in a psychiatric hospital. J. Psychiatr. Ment. Health Nurs..

[7] Lavis J.N., Robertson D., Woodside J.M., McLeod C.B., Abelson J. (2003). How can research organizations more effectively transfer research knowledge to decision makers?. Milbank Q..

[8] NSW Health NSW Health Smoke-Free Health Care Policy 2015. www0.health.nsw.gov.au/policies/pd/2015/pdf/PD2015_003.pdf.

[9] Zwar N., Richmond R., Borland R.H., Stillman S., Cunningham M., Litt J.C.B. (2005). Smoking cessation guidelines for Australian general practice. Aust. Fam. Physician.

[10] Freund M., Campbell E., Paul C., McElduff P., Walsh R.A., Sakrouge R., Wiggers J., Knight J. (2008). Smoking care provision in hospitals: A review of prevalence. Nicotine Tob. Res..

[11] Slattery C., Freund M., Gillham K., Knight J., Wolfenden L., Bisquera A., Wiggers J. (2015). Increasing smoking cessation care across a network of hospitals: An implementation study. Implement. Sci..

[12] Mullins R., Borland R. (1998). Changing the way smoking is measured among Australian adults: A preliminary investigation of Victorian data. Quit Eval. Stud..

[13] Richter K.P., Gibson C.A., Ahluwalia J.S., Schmelzle K.H. (2001). Tobacco use and quit attempts among methadone maintenance clients. Am. J. Public Health.

[14] Walsh R.A., Bowman J.A., Tzelepis F., Lecathelinais C. (2005). Smoking cessation interventions in Australian drug treatment agencies: A national survey of attitudes and practices. Drug Alcohol. Rev..

[15] Nagle A., Schofield M., Redman S. (1999). Australian nurses’ smoking behaviour, knowledge and attitude towards providing smoking cessation care to their patients. Health Promot. Int..

[16] Meshefedjian G.A., Gervais A., Tremblay M., Villeneuve D., O’Loughlin J. (2010). Physician smoking status may influence cessation counseling practices. Can. J. Public Health.

[17] Gunes G., Karaoglu L., Genc M.F., Pehlivan E., Egri M. (2005). University hospital physicians’ attitudes and practices for smoking cessation counseling in Malatya, Turkey. Patient Educ. Couns..

[18] Kurcer M.A., Simsek Z., Gunes G. (2005). Attitudes and practices of physicians, who have different smoking habits towards smoking cessation counselling in hospitals. Turk. J. Public Health.

[19] McCarty M.C., Hennrikus D.J., Lando H.A., Vessey J.T. (2001). Nurses’ attitudes concerning the delivery of brief cessation advice to hospitalized smokers. Prev. Med..

[20] Freund M.A., Campbell E.M., Paul C.L., Wiggers J.H., Knight J.J., Mitchell E.N. (2008). Provision of smoking care in NSW hospitals: Opportunities for further enhancement. N. S. W. Public Health Bull..

[21] Boomer M.J., Rissel C. (2002). An evaluation of a smoke free environment policy in two Sydney hospitals. Aust. Health Rev..

[22] Sørensen L.T. (2012). Wound healing and infection in surgery: The pathophysiological impact of smoking, smoking cessation, and nicotine replacement therapy: A systematic review. Ann. Surg..

[23] Sørensen L.T. (2012). Wound healing and infection in surgery. The clinical impact of smoking and smoking cessation: A systematic review and meta-analysis. Arch. Surg..

[24] (2011). Toolkit Economics of Tobacco: Assessment of the Economic Costs of Smoking.

[25] Nawfal A., Sewell M.D., Bhavikatt M., Gikas P.D. (2012). The effect of smoking on fracture healing and on various orthopaedic procedures. Acta Orthop. Belg..

[26] Warner D.O. (2007). Tobacco dependence in surgical patients. Curr. Opin. Anaesthesiol..

[27] Center for Disease Control and Prevention (2010). Vital signs: Nonsmokers’ exposure to secondhand smoke—United States, 1999–2008. Morb. Mortal. Wkly. Rep..

[28] Twardella D., Brenner H. (2005). Lack of training as a central barrier to the promotion of smoking cessation: A survey among general practitioners in Germany. Eur. J. Public Health.

[29] Kipo D.D. (2014). Agency-structure relation in social sciences: Reflections on policy implementation. Asian Soc. Sci..

[30] Campbell S., Pieters K., Mullen K.-A., Reece R., Reid R.D. (2011). Examining sustainability in a hospital setting: Case of smoking cessation. Implement. Sci..

[31] Nikathil S., Olaussen A., Gocentas R.A., Symons E., Mitra B. (2017). Workplace violence in the emergency department: A systematic review and meta analysis. Emerg. Med. Australas.

[32] Morphet J., Griffiths D., Plummer V., Innes K., Fairhall R., Beattie J. (2014). At the crossroads of violence and aggression in the emergency department: Perspectives of Australian emergency nurses. Aust. Health Rev..

[33] Robson D., Spaducci G., McNeill A., Stewart D., Craig T.J.K., Yates M., Szatkowski L. (2017). Effect of implementation of a smoke-free policy on physical violence in a psychiatric inpatient setting: An interrupted time series analysis. Lancet Psychiatry.

[34] Patterson P.B., Hawe P., Clarke P., Krause C., van Dijk M., Penman Y., Shiell A. (2009). The worldview of hospital security staff: Implications for health promotion policy implementation. J. Contemp. Ethnogr..

[35] Braun B.L., Fowles J.B., Solberg L.I., Kind E.A., Lando H., Pine D. (2004). Smoking-related attitudes and clinical practices of medical personnel in Minnesota. Am. J. Prev. Med..

[36] Gomm M., Lincoln P., Egeland P., Rosenberg M. (2002). Helping hospitalised clients quit smoking: A study of rural nursing practice and barriers. Aust. J. Rural Health.

[37] Li I.C., Lee S.Y.D., Chen C.Y., Jeng Y.Q., Chen Y.C. (2014). Facilitators and barriers to effective smoking cessation: Counselling services for inpatients from nurse-counsellors’ perspectives—A qualitative study. Int. J. Environ. Res. Public Health.

[38] DeLucia P.R., Ott T.E., Palmieri P.A. (2009). Performance in nursing. Rev. Hum. Factors Ergon..

[39] Sarna L., Bialous S.A., Zou X.N., Wang W., Hong J., Wells M., Brook J. (2016). Evaluation of a web-based educational programme on changes in frequency of nurses’ interventions to help smokers quit and reduce second-hand smoke exposure in China. J. Adv. Nurs..

[40] Rice V.H., Hartmann-Boyce J., Stead L.F. (2013). Nursing interventions for smoking cessation. Cochrane Libr..

[41] Perka E.J. (2011). Culture change in addictions treatment: A targeted training and technical assistance initiative affects tobacco-related attitudes and beliefs in addiction treatment settings. Health Promot. Pract..

[42] Guydish J., Ziedonis D., Tajima B., Seward G., Passalacqua E., Chan M., Delucchi K., Zammarelli L., Levy M., Kolodziej M. (2012). Addressing Tobacco Through Organizational Change (ATTOC) in residential addiction treatment settings. Drug Alcohol. Depend..

[43] Myung S.K., McDonnell D.D., Kazinets G., Seo H.G., Moskowitz J.M. (2009). Effects of Web- and computer-based smoking cessation programs: Meta-Analysis of randomized controlled trials. Arch. Intern. Med..

[44] Shahab L., McEwen A. (2009). Online support for smoking cessation: A systematic review of the literature. Addiction.

[45] Boyle R., Solberg L., Fiore M. (2011). Use of electronic health records to support smoking cessation. Cochrane Database Syst. Rev..

[46] Bonevski B., Magin P., Horton G., Foster M., Girgis A. (2011). Response rates in GP surveys: Trialling two recruitment strategies. Aust. Fam. Physician.

[47] Cook J.V., Dickinson H.O., Eccles M.P. (2009). Response rates in postal surveys of healthcare professionals between 1996 and 2005: An observational study. BMC Health Serv. Res..

[48] Cho Y.I., Johnson T.P., VanGeest J.B. (2013). Enhancing surveys of health care professionals. Eval. Health Prof..

