# Supplementary material for: Hospital Smoke-Free Policy: Compliance, Enforcement, and Practices. A Staff Survey in Two Large Public Hospitals in Australia
Source: Int J Environ Res Public Health. 2017 Nov 8;14(11):1358. doi: 10.3390/ijerph14111358 (PMC5707997; doi:10.3390/ijerph14111358)
Supplement: Supplementary file 1 [file ijerph-14-01358-s001.zip › Supplementary Table.pdf]

Supplementary Table S1. Survey items

Question	Response option
Perceived policy enforcement	
How often is the total smoking ban enforced at your hospital?	Always
	Often
	Sometimes
	Rarely
	Never
	Unsure
Staff enforcement on other staff	
If you see a staff member smoking on campus, how often do you:<br>- Ask them to stop smoking?<br>- Ask them to go outside the hospital premises for smoking?	Always
	Often
	Sometimes
	Rarely
	Never
	Unsure
	Not applicable
Staff enforcement on patients	
If you see a patient smoking on campus, how often do you:<br>- Ask them to stop smoking?<br>- Ask them to go outside the hospital premises for smoking?<br>- Offer them Nicotine Replacement Therapy instead of smoking?	Always
	Often
	Sometimes
	Rarely
	Never
	Unsure
	Not applicable
Perceived policy compliance	
How often do patients from your ward who smoke adhere to the smoking restrictions at the hospital?	Always
	Often
	Sometimes
	Rarely
	Never
	Unsure
How well are the hospital smoking restrictions adhered to by staff? That is, for staff members who smoke, how well do they adhere to the hospital no smoking policy?	Always
	Often
	Sometimes
	Rarely
	Never
	Unsure
Provision of smoking cessation care	
When talking to a patient who had identified as a current smoker, how often do you:<br>- Ask them about their current level of use<br>- Advise them to quit<br>- Assess their willingness to quit<br>- Assist them with a quit plan<br>- Refer them to specialist stop smoking services or counsellor<br>- Refer them to telephone Quitline<br>- Offer Nicotine Replacement Therapy	Always
	Most of the time
	Rarely
	Never

- Offer them other pharmacotherapies to help them quit<br>- Provide post-discharge help to quit<br>- Arrange follow-up with them either by yourself or another service	
In your hospital, how is the decision concerning whether or not to provide hospitalised patients who smoke with assistance to quit made?	Assistance is offered to every patient who smokes
	Assistance is offered on a patient-by-patient basis
	Assistance is offered only if the patient requests it
	Unsure

Supplementary Table S2. Sociodemographic characteristics of sample.

Variable		Total (n= 805) n (%)
Gender	Male	130 (20.1%)
	Female	518 (79.9%)
Age	Mean (SD)	42.45 (12.3)
Role at hospital	Intern	11 (1.4%)
	Resident (Junior resident, senior resident)	25 (3.1%)
	Registrar (Junior registrar, unaccredited registrar, accredited registrar, fellow)	57 (7.1%)
	Consultant	81 (10.1%)
	Division 1 nurse (registered)	405 (50.3%)
	Division 2 nurse (enrolled)	27 (3.4%)
	Other	199 (24.7%)
Employment	Full-time	482 (61.7%)
	Part-time	268 (34.3%)
	Casual	19 (2.4%)
	Other	12 (1.5%)
Time at hospital	Less than 12 months	49 (6.3%)
	1-3 years	105 (13.5%)
	4-6 years	129 (16.5%)
	7-9 years	102 (13.1%)
	10 or more years	395 (50.9%)
Smoking status	Smoker	36 (5.6%)
	Non-smoker	609 (94.4%)
Intentions to quit	Quit in the next 30 days	5 (13.9%)
	Quit in the next 6 months	8 (22.2%)
	Quit, but not in the next 6 months	6 (16.7%)
	Never quit/Unsure	17 (47.2%)
